# The integrated landscape of fatty acid metabolism subtypes reveals with prognostic and therapeutic relevance in pancreatic cancer

**DOI:** 10.3389/fgstr.2022.969533

**Published:** 2022-10-21

**Authors:** Peng Dai, Jing Feng, Yanyan Dong, Shujing Zhang, Xiaopeng Cui, Xueliang Qin, Shiming Yang, Daguang Fan

**Affiliations:** ^1^ Department of Hepato-Pancreatic-Biliary Surgery, Shanxi Provincial People’s Hospital, Taiyuan, China; ^2^ Department of Gastroenterology, Shanxi Provincial People’s Hospital, Taiyuan, China; ^3^ Department of Pathology, Shanxi Provincial People’s Hospital, Taiyuan, China; ^4^ Department of Digestive Endoscopy, Shanxi Provincial People’s Hospital, Taiyuan, China

**Keywords:** pancreatic cancer, fatty acid metabolism subtype, prognosis, immunotherapy, chemotherapy, lncRNA signature

## Abstract

**Background:**

Pancreatic Cancer (PAAD) is one of the most commonly diagnosed malignancies and the leading cause of cancer-related death worldwide. Aberrantly expressed long noncoding RNAs (lncRNAs) are involved in tumourigenesis of PAAD, and associated with the overall survival and tumor fatty acid metabolism in PAAD patients.

**Methods:**

The data on gene expression and corresponding clinical characteristics of PAAD patients in TCGA-PAAD (N=177) and GSE62452 (N=65) are taken from The Cancer Genome Atlas (TCGA) and Gene Expression Omnibus (GEO) databases. Consensus cluster analysis to identify distinct fatty acid metabolism subtypes in PAAD based on 62 fatty acid metabolism gene. The single sample GSEA (ssGSEA) algorithm was developed for evaluation of tumor infiltrating immune cells between fatty acid metabolism subtypes. As well, the R package “pRRophetic” was used to predict chemotherapeutic response in PAAD patients. Tumor Immune Dysfunction and Exclusion (TIDE) was used to predict immunotherapy response in PAAD patients. Univariate and multivariate Cox analysis were utilized to calculate the prognostic-related lncRNAs.

**Results:**

Totally, three fatty acid metabolism subtypes were obtained in PAAD based on 62 fatty acid metabolism gene. Kaplan-Meier (K-M) analysis showed that the overall survival rate of cluster3 group was significantly higher than the other two groups. Significant differences were seen between the three subtypes in immune cell infiltration characteristics and the immunotherapy response indicators, including Tumor mutational burden (TMB), immunophenoscore (IPS), and immune checkpoint molecules. The cluster1 group and cluster3 group were speculated to have the higher response to immunotherapy patients in cluster2 gains more benefit from chemotherapy than other groups. A 4-lncRNA signature was constructed based on the value of gene expression and regression coefficients which stratified patients into two risk groups. Patients in the higher-risk group had lower survival probabilities than those in the lower-risk group, based on the Kaplan-Meier analysis and Cox regression analysis. Receiver operating characteristic (ROC) curve analysis confirmed the predictive capability. In GO and KEGG analysis, genes in the high-risk group were linked to PAAD development.

**Conclusions:**

We constructed a signature that could predict prognosis of PAAD and provide certain theory guidance for novel therapeutic approaches of PAAD.

## Introduction

Pancreatic Cancer (PAAD) is one of the most typical causes of cancer mortality and remains a challenging issue globally (1). In recent years, the fatty acid metabolism has become a more closely studied topic in the field of cancer (2). Metabolic disorders, especially fatty acid metabolism disorders, are an important microenvironment for tumor pathogenesis (3–5). To improve personalized therapy for PAAD patients, reliable and robust new fatty acid metabolism key molecules are urgently needed.

In contrast with mRNA, LncRNA do not possess protein-coding abilities and are a diverse and abundant group of noncoding RNA transcripts (6, 7). The increasing evidence indicates that aberrant lncRNA expression plays a critical role in the development and progression of tumors, primarily through epigenetic transcriptional regulation of coding genes (8–10). In recent years, multiple studies have revealed that lncRNA can be involved in the inflammatory process and fatty acid metabolism (11, 12). For example, lncRNA lnc-HC regulated hepatic fatty acid metabolism through miR-130b-3p (13). The AMP-activated protein kinase α (AMPKα) pathway regulates the metabolism of lipid droplets in hepatic stellate cells through LncRNA H19 (14). LncRNA kcnq1ot1 promoted lipid accumulation and accelerates atherosclerosis *via* functioning as competitive endogenous RNA (ceRNA) (15). The results of these studies suggest that lncRNAs, which may have specific expression patterns during fatty acid metabolism in particular types of cancer, may serve as novel biomarkers with diagnostic and prognostic value.

In the present study, we evaluated the predictive role of fatty acid metabolism subtypes in chemotherapy and immunotherapy. Moreover, we constructed a prognostic signature based on four fatty acid metabolism-related lncRNAs, which may be a new and independent indicator of PAAD patients, and the robustness of our signature can be evaluated.

## Materials and methods

### Data source and preprocessing

**Figure 1:**
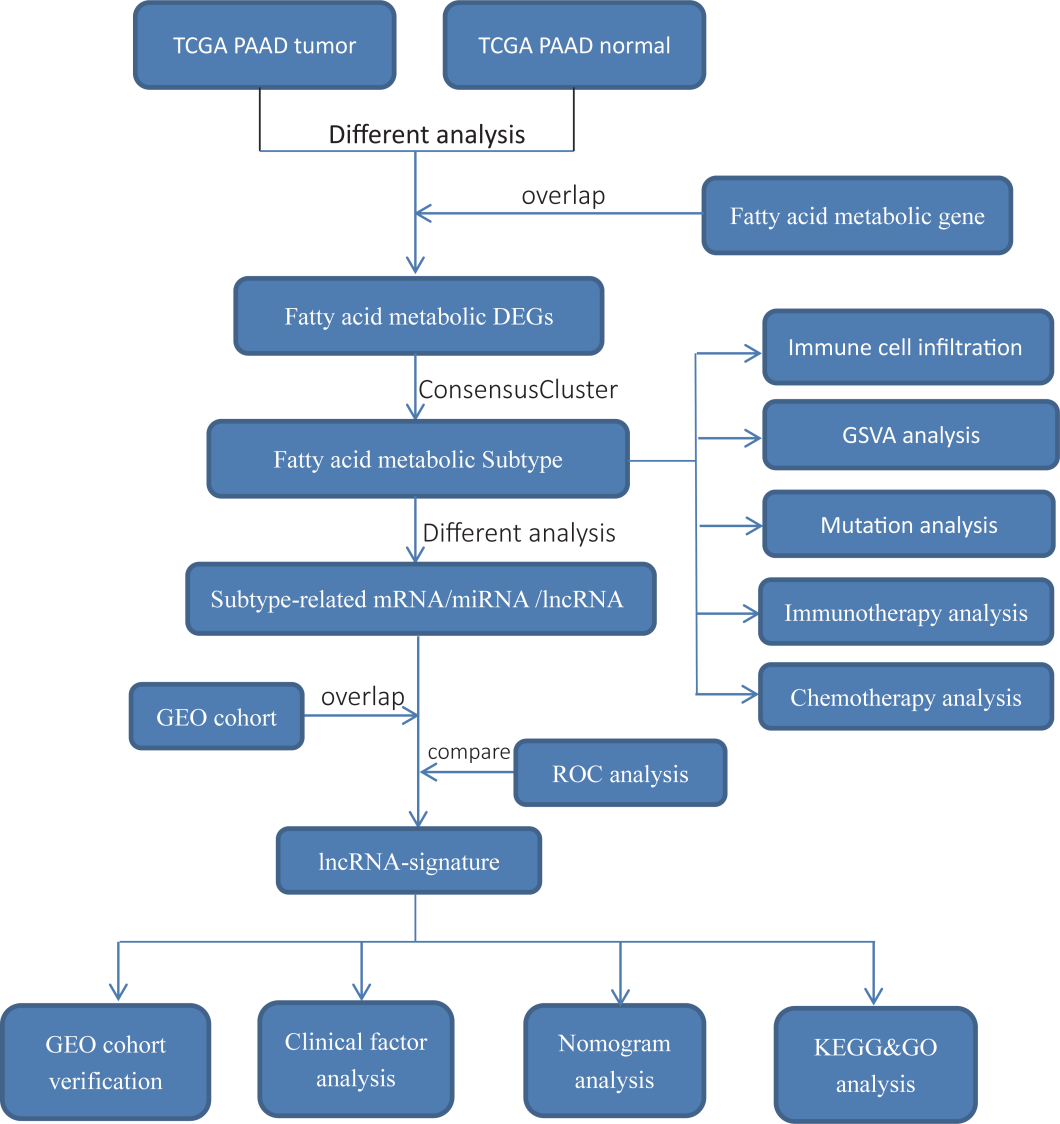
Overview of study design and analysis of fatty acid metabolism-related lncRNAs.

A flowchart of the study’s workflow was shown in **Figure 1**. This study included analysis of public gene-expression data were downloaded from The Cancer Genome Atlas (TCGA) and Gene Expression Omnibus (GEO). Fragments per kilobase per million (FPKM) and count expression data of PAAD as well as corresponding clinical information were downloaded from the UCSC Xena browser (http://xena.ucsc.edu/). The FPKM values were transformed into TPM values (transcript per million) to calculate the expression levels in different samples. In the further evaluation, patients with no survival information were removed. lncRNA expression profile was derived from the expression data using the lncRNA information in the GENECODE V22 data resource (https://www.gencodegenes.org/). miRNA expression data were downloaded from TCGA database. Furthermore, we only kept TCGA-PAAD samples that contain lncRNA, miRNA and mRNA expression data. In the present study, TCGA-PAAD datasets and GEO datasets (GSE62452) were used for training cohort and validation cohort, respectively. In the GEO cohort, patients with no survival information were removed (GSM1527113, GSM1527119, GSM1527121, GSM1527153 were removed). Meanwhile, the expression data were normalized by Z-score (standardized conversion) method, respectively. The fatty acid metabolism genes were obtained from GSEA database (http://www.broad.mit.edu/gsea). We performed Bioconductor R package “edgeR” to calculate differential expression fatty acid metabolism genes (DE fatty acid metabolism genes) based on count data of TCGA-PAAD normal samples and tumor samples. Differentially expressed fatty acid metabolism genes were determined using the cutoff thresholds of P< 0.05 and |log2 fold change| > 0.5. Mutation data was obtained from the TCGA Somatic Mutation database (http://tcga-data.nci.nih.gov/tcga/). Detailed patient characteristics of TCGA-PAAD were given in **Supplementary Table S1**.

### Consensus cluster of fatty acid metabolism gene

We performed the consensus cluster analysis to identify distinct fatty acid metabolism transcriptional regulation patterns based on the expression of DE fatty acid metabolism genes. The algorithm for consensus clustering determined the number of clusters and their stability. In order to ensure the classification’s stability, we performed 1000 repetitions of the above steps using the consensus clustering package “consensusclusterplus”. We performed bioconductor “limma” package to calculate differential expression mRNAs/lncRNAs/miRNAs between different clusters. Differentially expressed mRNAs/lncRNAs/miRNAs were determined using the cutoff thresholds of FDR< 0.05 and |log2 fold change| > 1.

### Evaluation of tumor infiltrating immune cells between fatty acid metabolism cluster

Using the single sample gene set enrichment analysis (ssGSEA) algorithm, we quantified the relative proportion of immune cells replete with tumor-infiltrating characteristics within the tumor microenvironment (TME). A set of biomarker genes for 28 types of immune cells was acquired from a past study. A total of 28 immune cell subtypes including MDSC, activated dendritic cell, macrophage, natural killer T cell, and regulatory T cell (16).

### Gene set variation analysis

Using “GSVA” R packages, we performed GSVA enrichment analysis to examine biological pathways between fatty acid metabolism clusters. The GSVA method is most commonly used to estimate the variation in pathway and biological process activity in a dataset of expression samples in a non-parametric and unsupervised manner (17). The gene sets for “Hallmark gene sets” were downloaded from MSigDB database for GSVA analysis. A p-value less than 0.05 was considered statistically significant.

### Prediction of chemotherapy response and immunotherapy response

By calculating half-maximal inhibitory concentration (IC50) of the samples using ridge regression, the R package “pRRophetic” was used to predict chemotherapeutic response in PAAD patients. Tumor Immune Dysfunction and Exclusion (TIDE) was used to predict patient response to immune checkpoint blockade (CTLA4 and PD1) therapy (18).

### Risk assessment model construction and prognostic survival analysis

We performed univariate Cox regression to calculate the prognostic fatty acid metabolism-related mRNAs/lncRNAs/miRNAs. The P-value less than 0.05 was considered as significance. The fatty acid metabolism-associated mRNAs/lncRNAs/miRNAs were subjected to univariate Cox regression to identify those associated with PAAD overall survival (OS). During the multiple stepwise regression analysis, we included only mRNAs/lncRNAs/miRNAs with statistical significance (P<0.05). Using the multivariate regression coefficients of mRNAs/lncRNAs/miRNAs expression, a risk assessment model was developed for the patients. Therefore, we computed the risk score by combining the expression values of lncRNAs/mRNAs/miRNAs included in the analysis, weighted by the linear regression model coefficients.


Risk score=Exp1∗Coe1+Exp2∗Coe2+Exp3∗Coe3+…Expi∗Coei

Using the PAAD risk assessment model, the patients were assigned to either a high- or low-risk group based on the calculated cutoff values calculated by the “survminer” R package. The Kaplan-Meier method was used to assess the effectiveness of OS among high-risk and low-risk patients. A P< 0.05 was considered significant under the log-rank test. Likewise, similar prognostic survival analyses were conducted on the validation cohort from GEO.

### Gene sets enrichment analysis

To investigate biological process differences between different studies, we conducted an GO (Gene Ontology) and KEGG (Kyoto Encyclopedia of Genes and Genomes) enrichment analysis using “clusterProfiler” R package. Adjusted P-value of less than 0.05 was considered statistically significant.

### Statistical analysis

The statistical analyses were performed using R (4.1.0) software, and P< 0.05 was considered statistically significant. The Kruskal-Wallis test was used for comparisons involving three or more factors.

## Results

### Fatty acid metabolism subtype had prognostic values in PAAD patients

**Figure 2:**
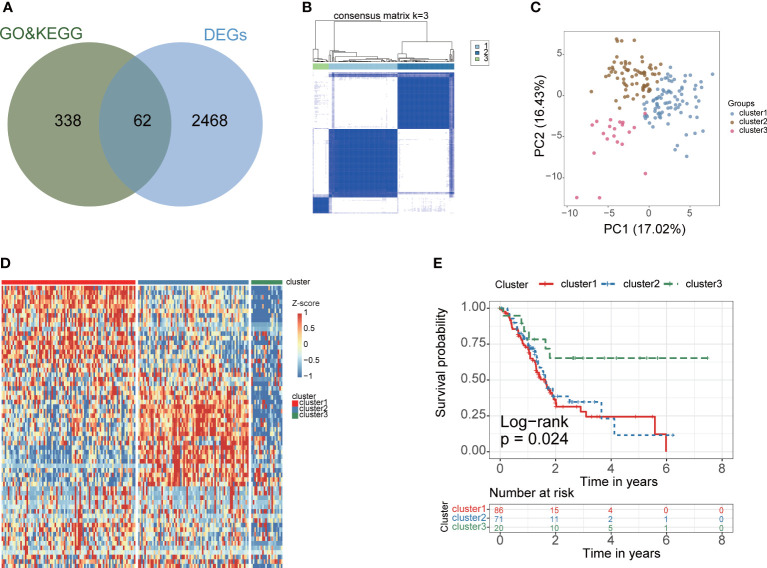
Construction of PAAD fatty acid metabolism subtype for PAAD. **(A)** The venn plot showed that 62 fatty acid metabolism-related genes were obtained in TCGA cohort. Green circles indicate fatty acid metabolism-related genes were download from KEGG and GO database, blue circles indicate differential expression gene between cancer and corresponding paracancerous tissues in TCGA cohort. **(B)** The optimal number of clusters (K=3) was determined, and the classification effect is the best. **(C)** PCA score plot. Blue dots represent cluster 1 patients, green dots represent cluster 2 patients, red dots represent cluster 3 patients. **(D)** The expression of 62 fatty acid metabolism-related genes between fatty acid metabolism subtypes. **(E)** K-M analysis of three fatty acid metabolism subgroup in the TCGA cohort.

The gene expression data and corresponding clinical data were downloaded from the TCGA and GEO database. Two eligible PAAD cohorts (GSE62452 (N=65) and TCGA-PAAD (N=177)) were integrated in our study for further analysis. We obtained 2530 differentially expressed genes (DEGs) between tumor and normal samples in TCGA cohort. Venn plot showed that 62 fatty acid metabolism genes were contained in DEGs (**Figure 2A**). According to consensus cluster analysis of 62 fatty acid metabolism genes, three clusters are the optimal number (**Figure 2B**). According to the clustering result, PAAD samples were clustered into cluster 1, 2 and 3 (N =86, 71 and 20, respectively). The PCA plot showed a clear grouping (**Figure 2C**). Heatmap showed the whole fatty acid metabolism genes expression between fatty acid metabolism subtypes (**Figure 2D**). The survival analysis demonstrated that the overall survival in the cluster3 group was markedly higher in contrast with the cluster1 group and cluster2 group, indicating that fatty acid metabolism mediated subtypes had prognostic values in PAAD patients (P=0.024) (**Figure 2E**).

### Significant differences of biological features in fatty acid metabolism subtypes

**Figure 3:**
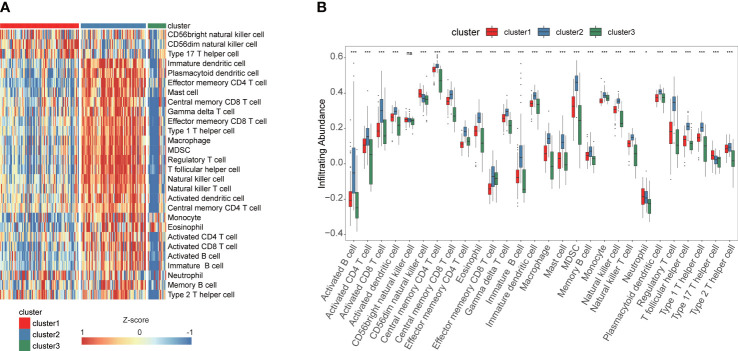
ssGSEA identified the relative infiltration of 28 immune cell types subpopulations with different fatty acid metabolism subtypes in TCGA cohort. **(A)** The relative infiltration of each cell type was normalized into Z-score. **(B)** The boxplot showed that 28 immune cell types in different fatty acid metabolism subtypes. ***P<0.001, *P<0.05, ns, not statistically significant.

**Figure 4:**
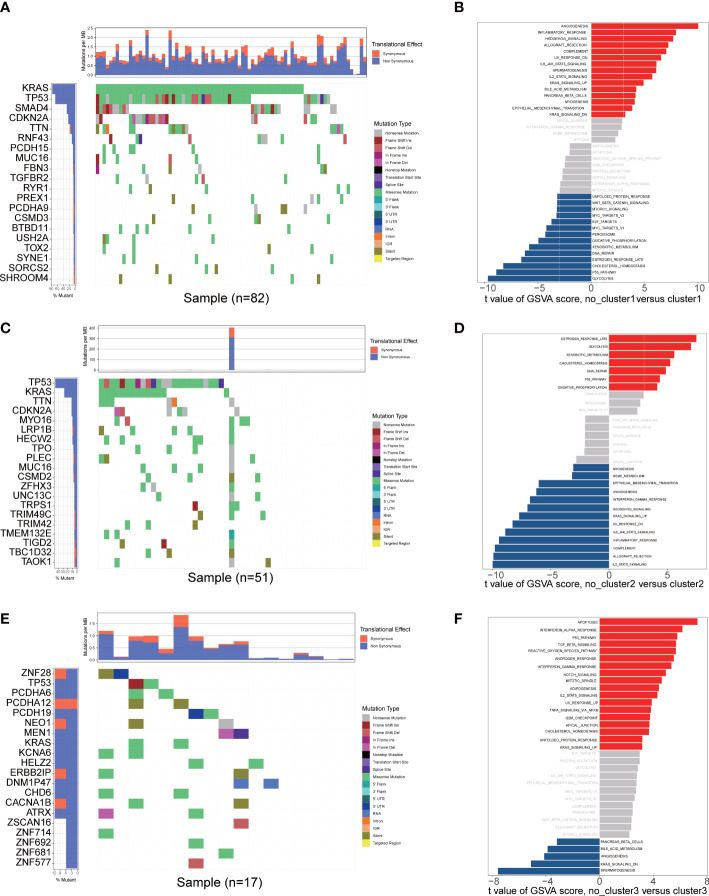
Mutational landscape and GSVA analysis. **(A, C, E)** Mutational landscape of gene in the three fatty acid metabolism subtypes. **(B, D, F)** The GSVA plot showed a differentially activated pathway between the three fatty acid metabolism subtypes. Each red bar showed an activated pathway, and each blue bar showed an inactivated pathway. (|t value| > 3 and *P<* 0.05).

The enrichment scores of diverse immune cell subpopulations were quantified in order to investigate the relationship between the fatty acid metabolism subtypes and tumor infiltrating immune cells by ssGSEA algorithm. According to the results, patients in the cluster2 group had a higher proportion of 28 immune cells activated compared to the cluster1 and cluster3 groups (P< 0.05) (**Figures 3A, B**). These results indicated that fatty acid metabolism played critical role in immune infiltration which might have significant clinical value in PAAD patients. The waterfall plot showed the gene mutation ratio in PAAD samples among fatty acid metabolism subtypes, and we determined several differentially mutation genes including in TP53 (P=6.273e-05, fisher exact test) and KRAS (P=7.131e-11, fisher exact test) (**Figures 4A, C, E**). Gene set variation analysis (GSVA) showed that Inflammatory response signaling were activated in the cluster1 group and cluster3 group, which was contrary to the results of the cluster2 group. In the cluster2 group, estrogen response late signaling was upregulated (**Figures 4B, D, F**).

### Different response of chemotherapy and immunotherapy in fatty acid metabolism subtypes

**Figure 5:**
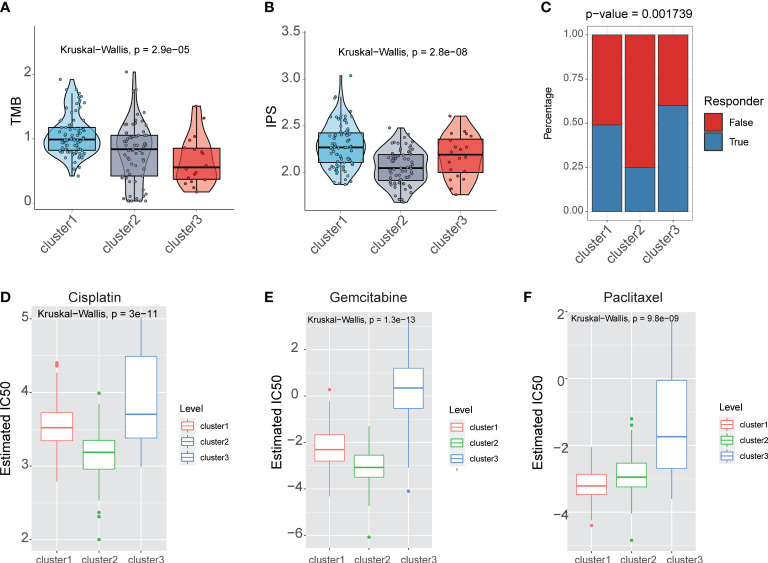
Fatty acid metabolism subtypes reveals with therapeutic relevance. **(A)** The boxplot showed that TMB in different fatty acid metabolism subtypes. **(B)** The boxplot showed that IPS in different fatty acid metabolism subtypes. **(C)** The barplot showed that immunotherapy response in different fatty acid metabolism subtypes. The IC50s of three chemotherapeutic agents [Cisplatin **(D)**, Gemcitabine **(E)**, and Paclitaxel **(F)**] in different fatty acid metabolism subtypes.

The analysis of the TMB pattern demonstrated that the cluster1 group had the highest TMB, the cluster2 group had intermediate TMB, and cluster3 had the lowest TMB (P = 2.9e-05) (**Figure 5A**). For Immunophenoscore (IPS) to determine the tumor immunogenicity and predict response to ICI therapy in multi types of tumors, we utilized IPS to investigate the immune response among the fatty acid metabolism subtypes. The result showed that the cluster1 and cluster3 group had the higher IPS, but the cluster2 group had the lower IPS (P = 2.8e-08) (**Figure 5B**), which suggested that the patients in cluster1 and cluster3 group might benefit from ICI therapy. We applied TIDE to predict the response of patients to immune checkpoint blockade (CTLA4 and PD1 therapy). TIDE analysis result also showed that cluster1 and cluster3 group might benefit from ICI therapy (P = 0.001739) (**Figure 5C**).

We performed the R package “pRRophetic” to estimate the response of chemotherapy drugs in three fatty acid metabolism subtypes. In contrast with patients in the cluster1 and cluster3 group, patients in the cluster2 group had lower IC50 in three chemotherapy drugs: Cisplatin, Gemcitabine, and Paclitaxel (**Figures 5D-F**).

### Identification of fatty acid metabolism related lncRNAs/mRNAs/miRNAs

We identified differentially expressed lncRNAs/mRNAs/miRNAs, i.e., fatty acid metabolism related lncRNAs/mRNAs/miRNAs, between three fatty acid metabolism subtypes. Results of differential expression analysis showed that 294 different expression lncRNA between three subtypes, 3737 different expression mRNA between three subtypes, 86 different expression miRNA between three subtypes. Then, we identified the intersection of differentially expressed lncRNAs/mRNAs between the TCGA and GEO datasets.

### Analysis of fatty acid metabolism related lncRNAs/mRNAs/miRNAs as prognostic biomarkers

A total of 143 fatty acid metabolism related mRNAs were analyzed *via* univariate cox regression. A total of 8 fatty acid metabolism related miRNAs were analyzed *via* univariate cox regression. A total of 12 fatty acid metabolism related lncRNAs were analyzed *via* univariate cox regression. The lncRNAs/mRNAs/miRNAs had significant prognostic significance (P<0.05) in univariate Cox proportional hazards regression analysis. Using multivariable Cox regression analysis on these selected lncRNAs, we finally obtained four lncRNAs, namely, (LINC01559, PART1, UCA1, FAM83A-AS1) (**Supplementary Table S2**). Using multiple stepwise regression analyses, a risk score was developed as follows:


$$\begin{array}{l} Risk\;score=0.1123\times \exp(LINC01559)-0.5301\times exp(PART1)+\\ \;0.1290\times \exp(UCA1)+0.1204\times exp(FAM83A-AS1) \end{array}$$

**Figure 6:**
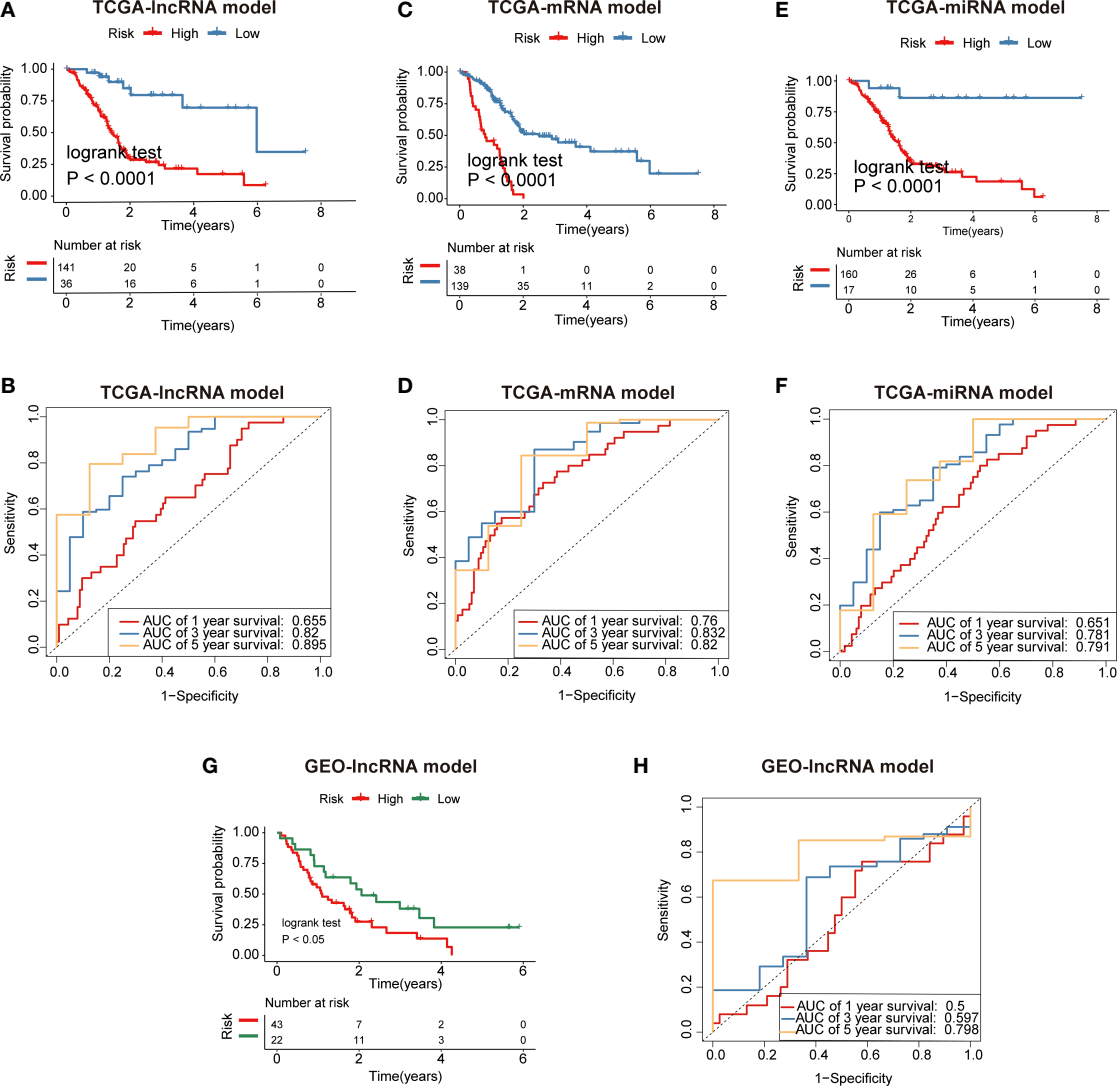
Identification of fatty acid metabolism-related lncRNA/mRNA/miRNA prognostic signature for PAAD. **(A)** Patients in the high-risk group (red) exhibited worse overall survival (OS) than those in the low-risk group (blue) in TCGA cohort (lncRNA model). **(B)** The receiver operator characteristic (ROC) curves to predict the sensitivity and specificity of 1-, 3-, and 5-years survival according to the 4-lncRNA signature derived risk scores in TCGA cohort. **(C)** Patients in the high-risk group (red) exhibited worse overall survival (OS) than those in the low-risk group (blue) in TCGA cohort (mRNA model). **(D)** The receiver operator characteristic (ROC) curves to predict the sensitivity and specificity of 1-, 3-, and 5-years survival according to the 4-lncRNA signature derived risk scores in TCGA cohort (mRNA model). **(E)** Patients in the high-risk group (red) exhibited worse overall survival (OS) than those in the low-risk group (blue) in TCGA cohort (miRNA model). **(F)** The receiver operator characteristic (ROC) curves to predict the sensitivity and specificity of 1-, 3-, and 5-years survival according to the 4-lncRNA signature derived risk scores in TCGA cohort (miRNA model). **(G)** Patients in the high-risk group (red) exhibited worse overall survival (OS) than those in the low-risk group (green) in GEO cohort (lncRNA model). **(H)** The receiver operator characteristic (ROC) curves to predict the sensitivity and specificity of 1-, 3-, and 5-years survival according to the 4-lncRNA signature derived risk scores in GEO cohort (lncRNA model).

**Figure 7:**
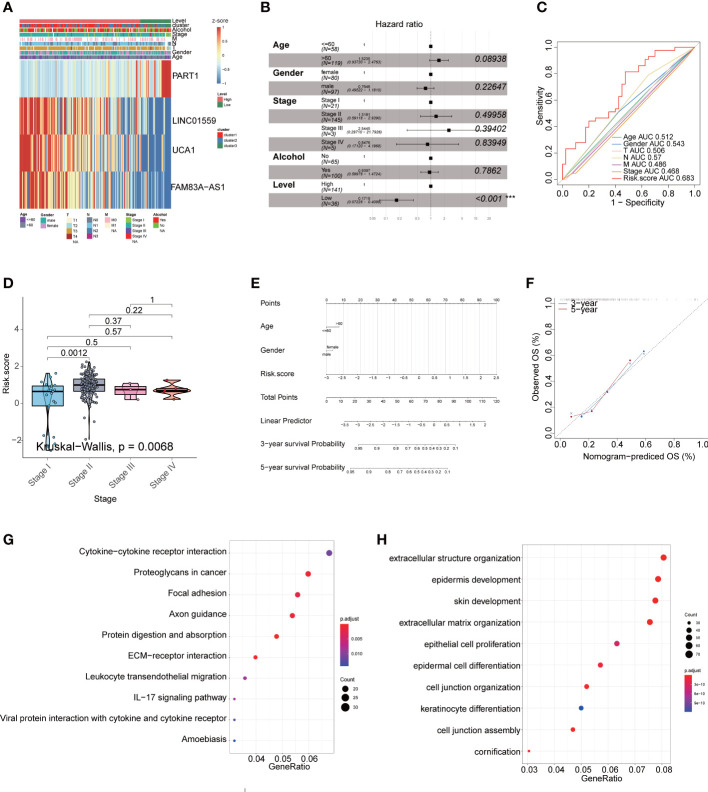
The lncRNA signature as an independent prognostic factor in PAAD. **(A)** The expression of four lncRNAs in TCGA cohort. **(B)** Multivariate Cox regression analyses of the association between clinic pathological factors and OS of PAAD patients ***P<0.001. **(C)** The receiver operator characteristic (ROC) curves to predict the sensitivity and specificity of clinic pathological factors and 4-lncRNA signature derived risk scores in PAAD patients. **(D)** The boxplot showed that risk score between stage. **(E)** The nomogram consists of the clinical features (age, gender), and risk score. The variable scores were summed to give the total points, and the total point line was shown at the bottom of the nomogram. **(F)** The calibration plots were established to compare the proposed nomogram with an ideal model. The KEGG **(G)** and GO **(H)** enriched gene pathways/functions in distinct fatty acid metabolism risk signature groups in TCGA PAAD patients.

Furthermore, we constructed mRNA/miRNA prognostic model using similar approach as above. The R package survminer calculated a cutoff value of 0.4629 for the low-risk and high-risk groups. Our data demonstrated that the overall survival (OS) rate in the low-risk group was markedly higher in contrast with the high-risk group (**Figure 6A**). In addition, ROC curves were used to evaluate the predictive value of the risk model, and the AUC for three- and five-year survival was 0.82 and 0.895, respectively. (**Figure 6B**). Compared with the mRNA prognostic model (AUC for three- and five-year survival was 0.832 and 0.82)(**Figures 6C, D**) and miRNA prognostic model (AUC for three- and five-year survival was 0.781 and 0.791) (**Figures 6E, F**), the lncRNA prognostic model (AUC for three- and five-year survival was 0.82 and 0.895) had better predictive power for survival in the TCGA-PAAD cohort. The validation cohort from GEO was also stratified into high- or low-risk groups in accordance with the same formula used to stratify the validation cohort (**Figures 6G, H**). The results indicated that a higher risk score was also associated with a poor OS. Heatmap analysis was used to visualize the expression of four lncRNAs in PAAD patient samples. (**Figure 7A**).

### The fatty acid metabolism-related lncRNA signature is an independent prognostic indicator

In multivariate Cox regression, the four lncRNAs related to fatty acid metabolism were analyzed for their prognostic value independently of clinicopathological indicators, such as age, gender, and pathological stage in PAAD patients. We found that the hazard ratio (HR) of risk score and 95% CI were 0.1719 (95% CI: 0.07228-0.4089) in multivariate Cox regression assessment (P< 0.001) (**Figure 7B**). When compared with the classic risk factor pathological stage (AUC = 0.468), the risk score (AUC = 0.683) had a better predictive power for survival in the TCGA-PAAD cohort (**Figure 7C**), which suggests that the lncRNA signature are independent PAAD prognostic factors. The boxplot showed the risk score were different between stage (P =0.0068) (**Figure 7D**). We then examined the clinical applications of the 4-lncRNAs signature with the nomogram. Nomograms are enumerated according to the algorithm for all variables significant (age, gender, and risk score) in the multivariate analysis. A noogram showing the probability of OS at 3 and 5 years was constructed by summing the variable scores and measuring the total point line, which was shown at the bottom of the nomogram (**Figures 7E**). As a result, the calibration plots suggested that in comparison with an ideal model, the proposed nomogram performed similarly (**Figures 7F**).

### Functional annotation related to the two risk groups

We conducted GO and KEGG enrichment analyses between the high-risk and low-risk groups to identify potential biological functions and pathways associated with the risk signature. We found that several pathways linked to inflammatory and cell proliferation in carcinoma including regulation of IL−17 signaling pathway, epithelial cell proliferation, and so on (**Figures 7G, H**).

## Discussion

PAAD is one of the most commonly diagnosed malignancies and is considered a leading cause of cancer-related deaths worldwide (19, 20). The influence of accumulated lipid due to fatty acid metabolism abnormalities on the tumor-microenvironment may also account for the poor prognosis (21–23). Therefore, it is urgent to explore potential mechanisms and reliable novel biomarkers for identifying and predicting the fatty acid metabolism of PAAD.

In our research, TCGA-PAAD cohort was classified into three clusters by unsupervised clustering of the gene expression of fatty acid metabolism, and there was a significant difference in survival between groups. Furthermore, we found several immune cell infiltration differences and mutated genes between three fatty acid metabolism subtypes. Subsequently, the DElncRNA/DEmiRNA/DEmRNA between the three clusters were specific genes regulating the level of fatty acid metabolism, and these genes may be directly or indirectly involved in the fatty acid metabolism process. In order to better evaluate the prognosis and treatment of PAAD patients individually, we screened these DElncRNA/DEmiRNA/DEmRNA through a risk model and, constructed a fatty acid metabolism signature, and verified the validity and applicability of the fatty acid metabolism signature.

Several previous studies have shown a correlation between fatty acid metabolism and cancer progression and treatment (24, 25), as well as immunity (26). Fatty acids secreted into the microenvironment affect infiltrating immune cell function and phenotype. Abnormal lipid metabolism, such as increased fatty acid oxidation and lipid *de novo* synthesis, can provide tumors with a survival advantage against chemotherapy and radiation therapy and alleviate cellular stress involved in the metastatic cascade. *De novo* fatty acid synthesis is involved in the activation process of T cells (27–29), and T cells can also degrade fatty acids as energy sources through β-oxidation like other cells. Fatty acid oxidation is related to CD8+ memory T cells, CD4+ regulatory T cells and other cells (30). In addition, proliferating B cells require monounsaturated FAs (MUFAs) to maintain mitochondrial metabolism and mTOR activity and protect against excessive autophagy and endoplasmic reticulum stress (26). In this study, the prognosis of cluster 3 was better than that of cluster 1 and cluster 2, and there were differences in immune cell infiltration such as T cells and B cells, between the three clusters, which may be related to their prognosis. And previous studies have shown that a high-fat diet leads to increased fatty acid uptake by tumor cells but not by tumor-infiltrating CD8 T cells. This imbalance in fatty acid distribution impairs CD8 T cell infiltration and function, implying that metabolically improved interventions for tumor immunotherapy can be exploited (31).

Several lncRNA models have been developed to predict the prognosis of PAAD patients. Such as, Liu et al. developed a m5C-Related lncRNA signature optimizes prediction of prognostic and immune responses in pancreatic cancer (32). However, few studies have investigated the fatty acid metabolism-related lncRNA signature associated with the prognosis of PAAD patients. In this study, we identified four fatty acid metabolism-associated lncRNA and developed a fatty acid metabolism related signature for predicting the prognosis of PAAD patients. Four lncRNAs were identified as a novel fatty acid metabolism signature by both univariate and multivariate Cox regression analysis, namely, LINC01559, PART1, UCA1, FAM83A-AS1. UCA1 was found to contribute to anti-cancer drug resistance and tumor cell invasion (33, 34).

However, our study was limited by several limitations. First is the immune-based therapies data of PAAD patients were not available now. The results of TIDE and IPS not very accurate. More validation datasets of received immunotherapy are needed to verify the stability of fatty acid metabolism subtypes. Another pitfall is that it relied exclusively on data from public databases, and the gene signature was mostly identified retrospectively. Because of this, case selection bias may influence the results. Further, the specific function and mechanism of the four lncRNAs in PAAD remains unclear and their expression profiles and clinical validation in the patients of prospective studies are inconclusive as well. In the future, we need to identify and incorporate more potential markers into our prediction model.

In conclusion, this study constructed a fatty acid metabolism subtypes by screening the fatty acid metabolism gene. This subtype can be used to assess the fatty acid metabolism level of PAAD patients, and can be use and to evaluate the immunotherapy and prognosis of PAAD patients. Compared with ROC in mRNA model and miRNA model, we constructed a lncRNA-fatty acid metabolism signature to assess prognosis in PAAD patients. However, more evidence is still needed to verify.

## Data availability statement

The original contributions presented in the study are included in the article/**Supplementary Material**. Further inquiries can be directed to the corresponding author.

## Author contributions

Conceptualization: PD, JF. Resources: YD, SZ. Data curation: XC. Software: XQ. Formal analysis: PD. Supervision: SY, DF. Methodology: PD. Writing-original draft: PD. Writing-review and editing: DF. All authors contributed to the article and approved the submitted version.

## Conflict of interest

The authors declare that the research was conducted in the absence of any commercial or financial relationships that could be construed as a potential conflict of interest.

## Publisher’s note

All claims expressed in this article are solely those of the authors and do not necessarily represent those of their affiliated organizations, or those of the publisher, the editors and the reviewers. Any product that may be evaluated in this article, or claim that may be made by its manufacturer, is not guaranteed or endorsed by the publisher.
